# Supplementary figures and images for: Open-source low-cost cardiac optical mapping system
Source: PLoS One. 2022 Mar 31;17(3):e0259174. doi: 10.1371/journal.pone.0259174 (PMC8970595; doi:10.1371/journal.pone.0259174)

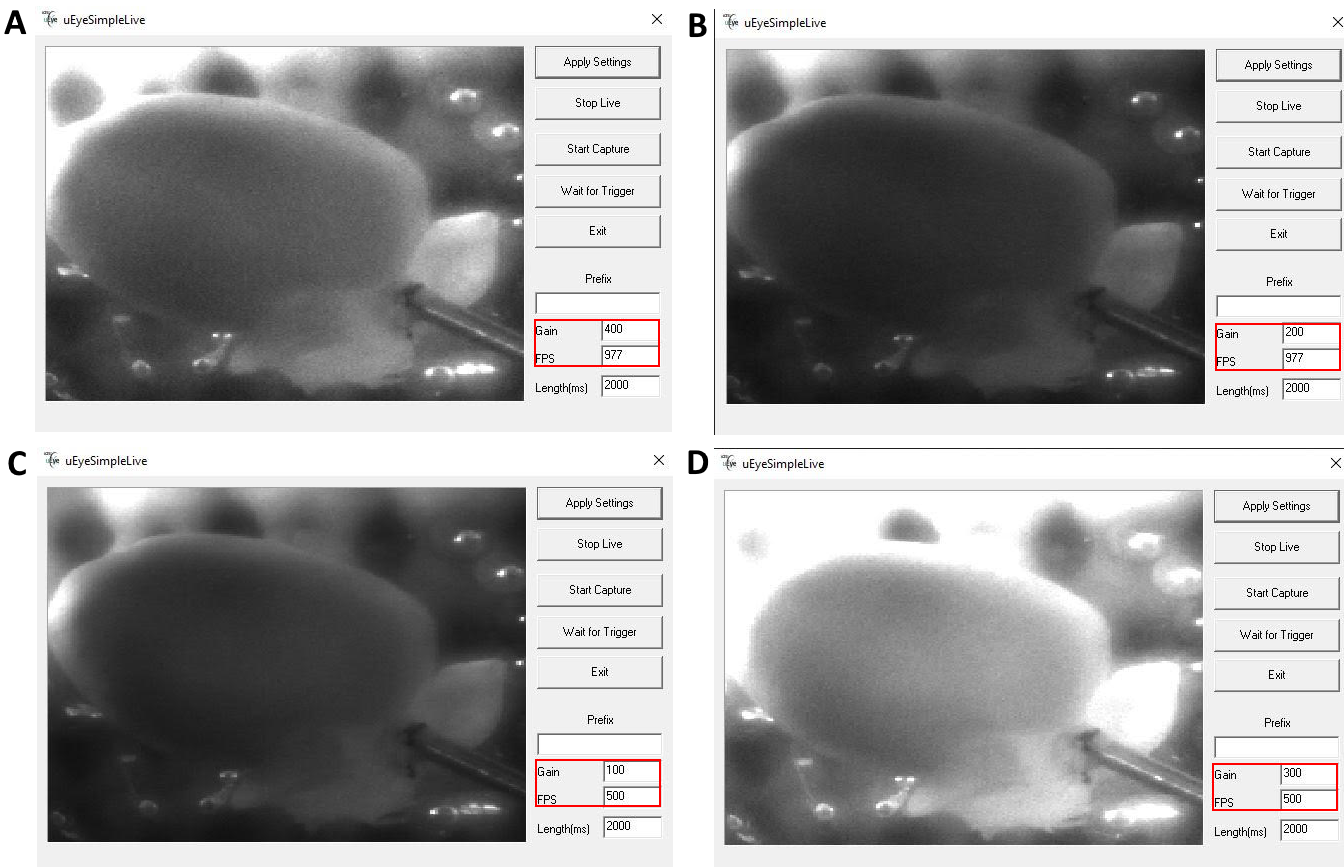

Supplement: S1 Fig — Screenshots of image acquisition software. Real-time viewfinder feed allows the user to adjust settings when signal amplitude is too low (B,C) or oversaturating (D). (TIF) [file pone.0259174.s001.tif]

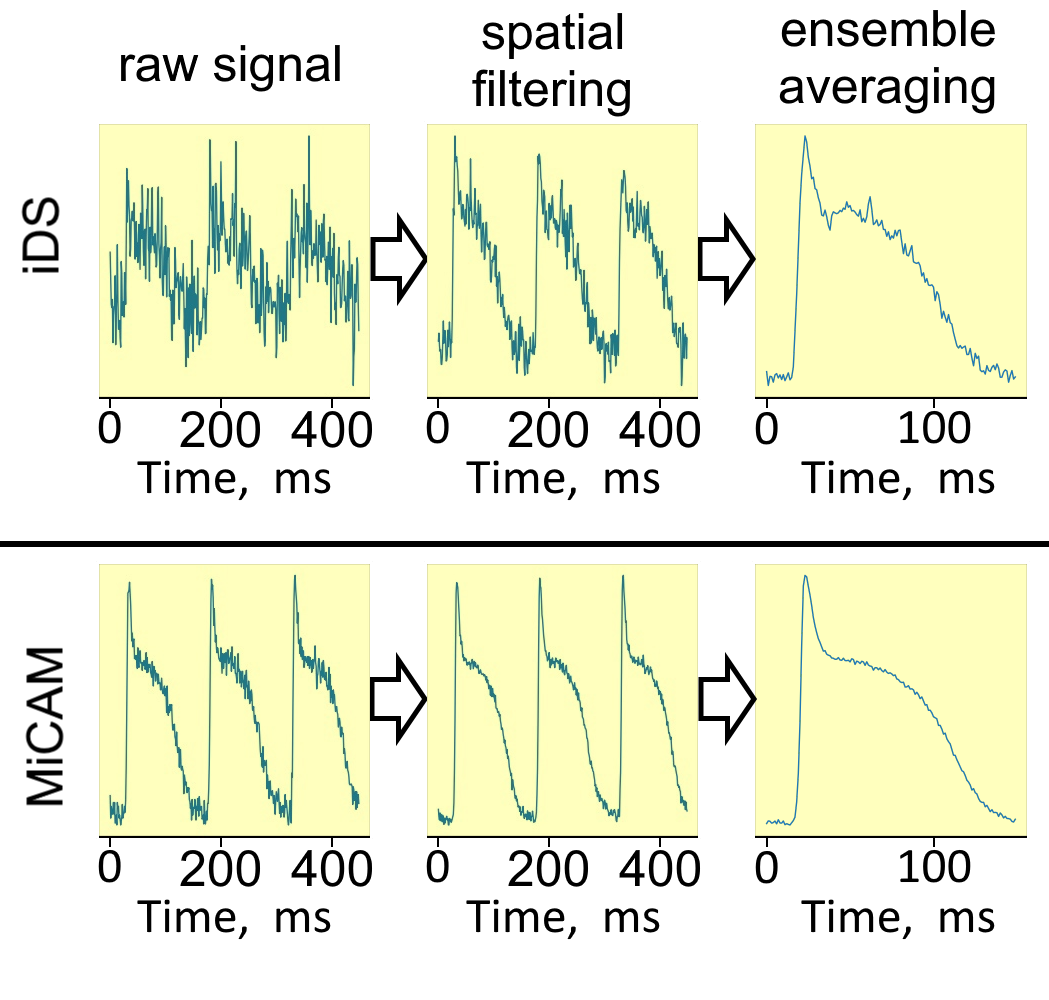

Supplement: S2 Fig — Samples of the signal from a single pixel after different steps of conditioning. (TIF) [file pone.0259174.s002.tif]

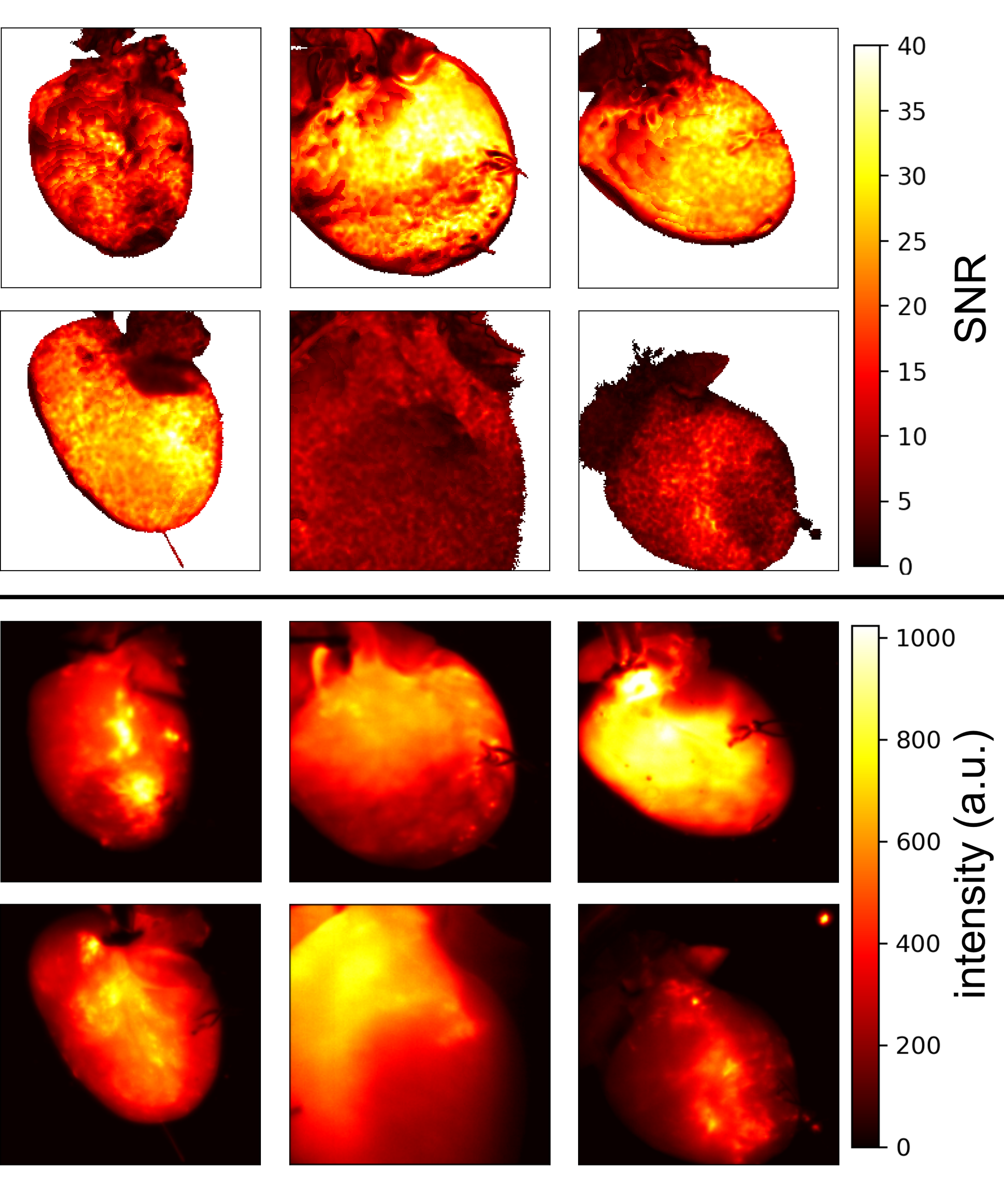

Supplement: S3 Fig — SNR maps and intensity maps for conditioned recordings of 6 mouse hearts recorded with iDS camera at PCL = 150 ms. (TIF) [file pone.0259174.s003.tif]
