# Supplementary material for: Open-source low-cost cardiac optical mapping system
Source: PLoS One. 2022 Mar 31;17(3):e0259174. doi: 10.1371/journal.pone.0259174 (PMC8970595; doi:10.1371/journal.pone.0259174)
Supplement: S1 Table — (DOCX) [file pone.0259174.s004.docx]

**S1 Table. Component prices in the MiCAM Ultimate-L system and the system presented in this study.**

| iDS | MiCAM |
| --- | --- |
| Camera: UI-3130CP M.GL. R2 ~$600 | Camera and computer: MiCAM Ultimate-L - $90,000 |
| Computer: ~$900 |  |
| Prizmatix UHP-Mic-LED-520 $3,000 | Prizmatix UHP-Mic-LED-520 $3,000 |
| Pentax C60607KP ~$150 | Nikkor 50mm f/1.2 lens ~$725 |
| Thorlabs 650nm long-pass filter ~$80 | Thorlabs 650nm long-pass filter ~$80 |
| Total: $4,730 | Total: $94,000 |
