# Supplementary material for: Open-source low-cost cardiac optical mapping system
Source: PLoS One. 2022 Mar 31;17(3):e0259174. doi: 10.1371/journal.pone.0259174 (PMC8970595; doi:10.1371/journal.pone.0259174)
Supplement: S1 Text — (PDF) [file pone.0259174.s005.pdf]

Software links

iDS camera acquisition software: *https://github.com/humanphysiologylab/ueyemappingWin*

Rhythm_1.2: *https://github.com/optocardiography/Rhythm-1.2*

Scripts used to analyze optical mapping recordings:

https://github.com/drybashlykov/ids_paper_scripts

Data link

DOI:10.5281/zenodo.5557828

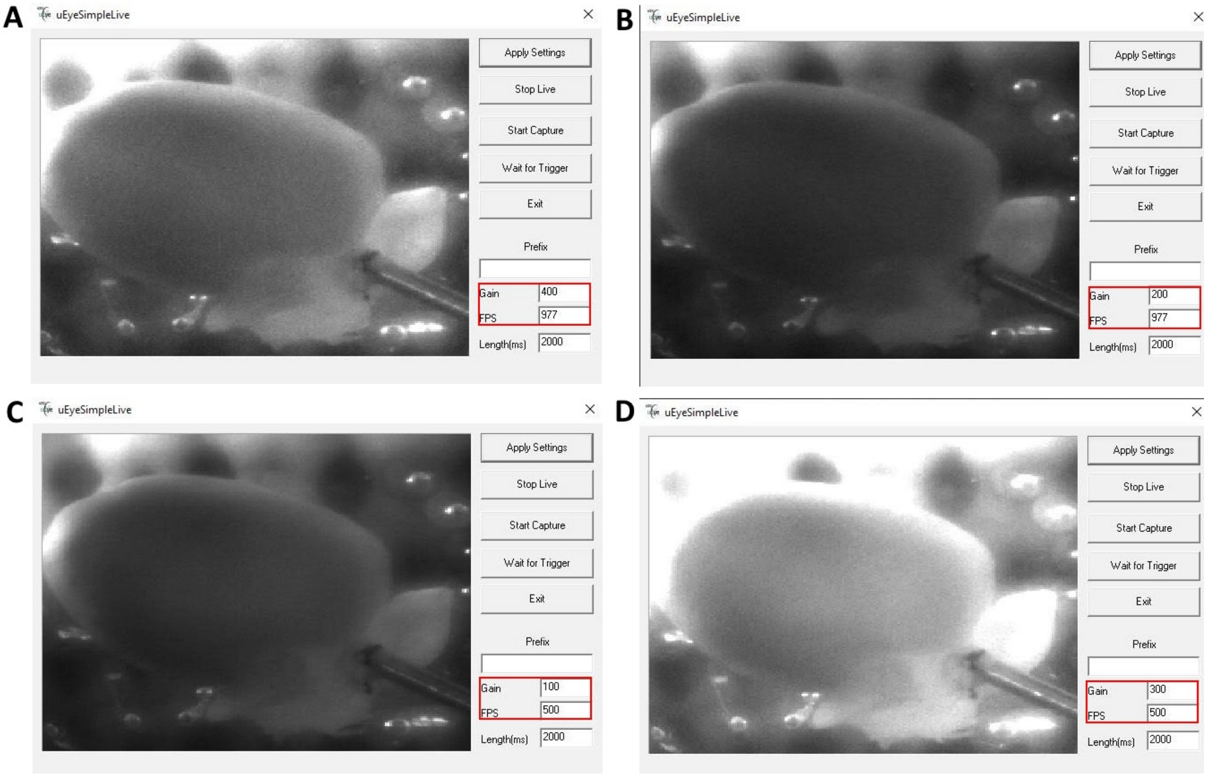

S1 Fig. Interactive image acquisition software. Screenshots of image acquisition software. Real-time viewfinder feed allows the user to adjust settings when signal amplitude is too low (B,C) or oversaturating (D).

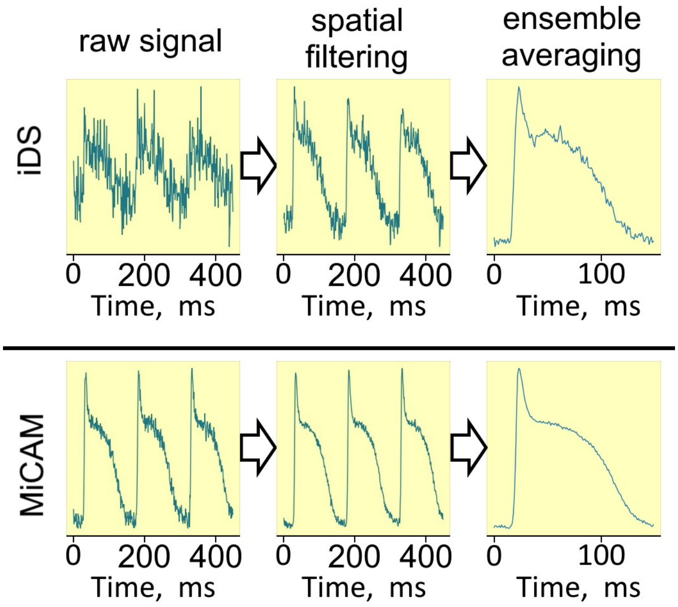

S2 Fig. Signal conditioning steps. Samples of the signal from a single pixel after different steps of conditioning.

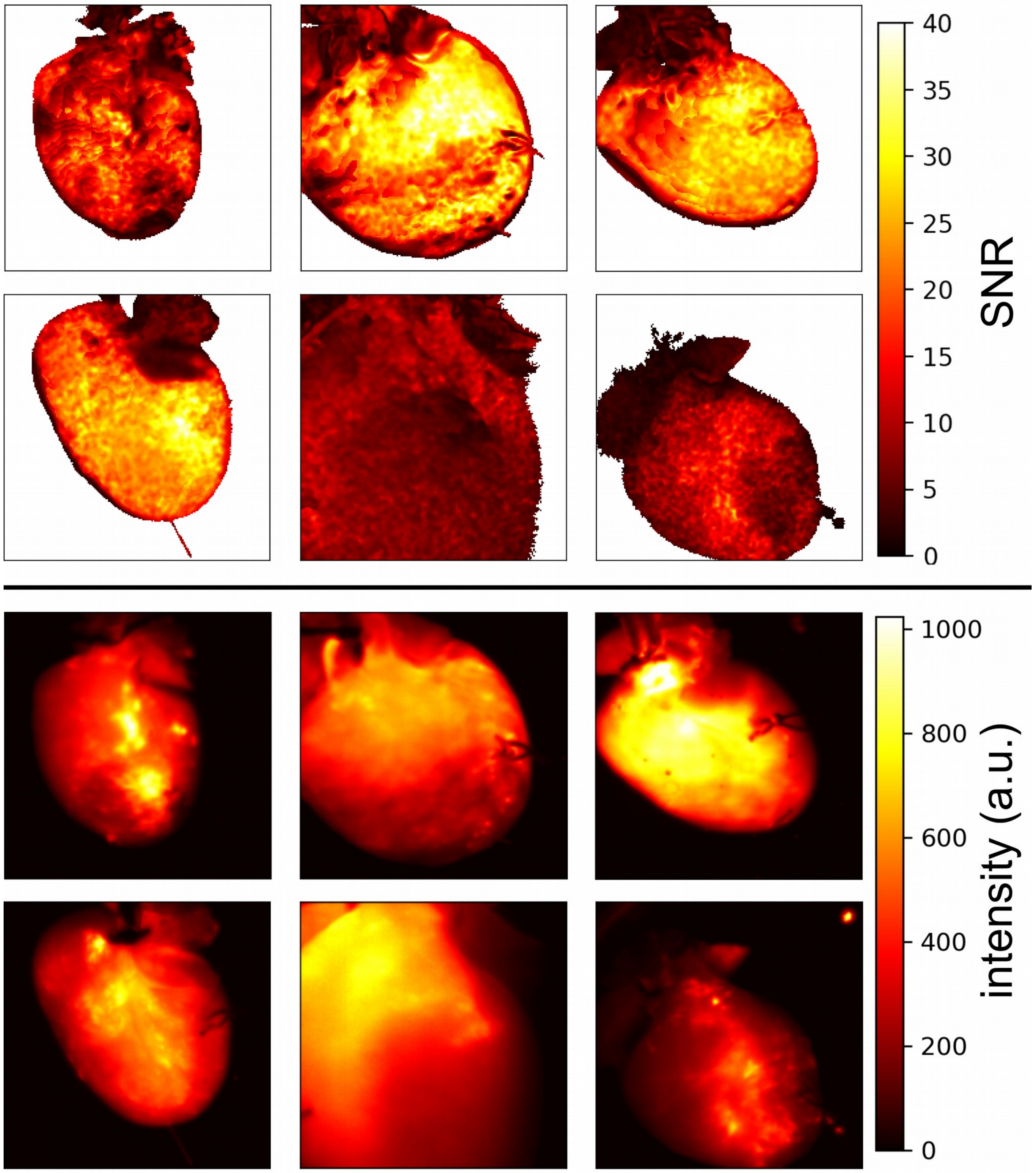

S3 Fig. **SNR and intensity maps.** SNR maps and intensity maps for conditioned recordings of 6 mouse hearts recorded with iDS camera at PCL = 150 ms.

S1 Table. Component prices in the MiCAM Ultimate-L system and the system presented in this study.

iDS	MiCAM
Camera: UI-3130CP M.GL. R2 ~$600	Camera and computer: MiCAM Ultimate-L - $90,000
Computer: ~$900	
Prizmatix UHP-Mic-LED-520 $3,000	Prizmatix UHP-Mic-LED-520 $3,000
Pentax C60607KP ~$150	Nikkor 50mm f/1.2 lens ~$725
Thorlabs 650nm long-pass filter ~$80	Thorlabs 650nm long-pass filter ~$80
Total: $4,730	Total: $94,000
